# A large helmet-shaped proliferating trichilemmal tumor of the scalp: Is definitive radiotherapy the treatment? A case report

**DOI:** 10.1186/s43046-019-0007-y

**Published:** 2019-12-11

**Authors:** Haresh Kunhi Parambeth, Nava Udhayam, Shipra Agarwal, Subhash Gupta, Prashanth Giridhar, Gour Kishor Rath

**Affiliations:** 1 Department of Radiation Oncology, All India Institute of Medical Sciences, New Delhi, 110029 India; 2 Department of Pathology, All India Institute of Medical Sciences, New Delhi, India

**Keywords:** Proliferating trichilemmal tumor, Radiotherapy, Radical

## Abstract

**Background:**

Benign proliferating trichilemmal tumors (PTTs) are a rare entity that arises from the outer root sheath of a hair follicle. They range from a benign PTT that recurs locally to the more aggressive malignant PTT that, in addition to recurring locally, has the potential for metastatic spread. However, as a group, PTTs are slow growing and amenable to surgery. To the best of our knowledge, radical radiotherapy without surgery has been used in only one case in an elderly male patient with good oncological and cosmetic results.

**Case presentation:**

We present a case of a young unmarried female with a disfiguring PTT of the scalp not amenable to surgery treated successfully with radiotherapy providing good cosmesis. Volumetric modulated arc therapy was used to treat this patient with a dose of 50 Gy in 25 fractions over 5 weeks. A theoretical risk of malignant transformation was explained to the patient. The patient has maintained good cosmesis over the last 12 months with no signs of re-growth.

**Conclusion:**

In patients with PTT not amenable to surgery, radiotherapy may be an effective alternative providing local control and good cosmesis.

## Background

Benign proliferating trichilemmal tumor (PTT) is a rare tumor that arises from the outer root sheath of a hair follicle [1]. It is usually slow growing and amenable to surgery. Radiotherapy is used as an adjuvant modality after surgery in selected cases [2] or as a palliative modality in metastatic disease. To the best of our knowledge, radical radiotherapy without surgery has been used in only one case of the malignant variant of PTT (MPTT) in an elderly male patient with good oncological and cosmetic results [1]. We here present a case of a young unmarried female with a disfiguring PTT of scalp treated with radiotherapy.

## Case presentation

**Fig. 1:**
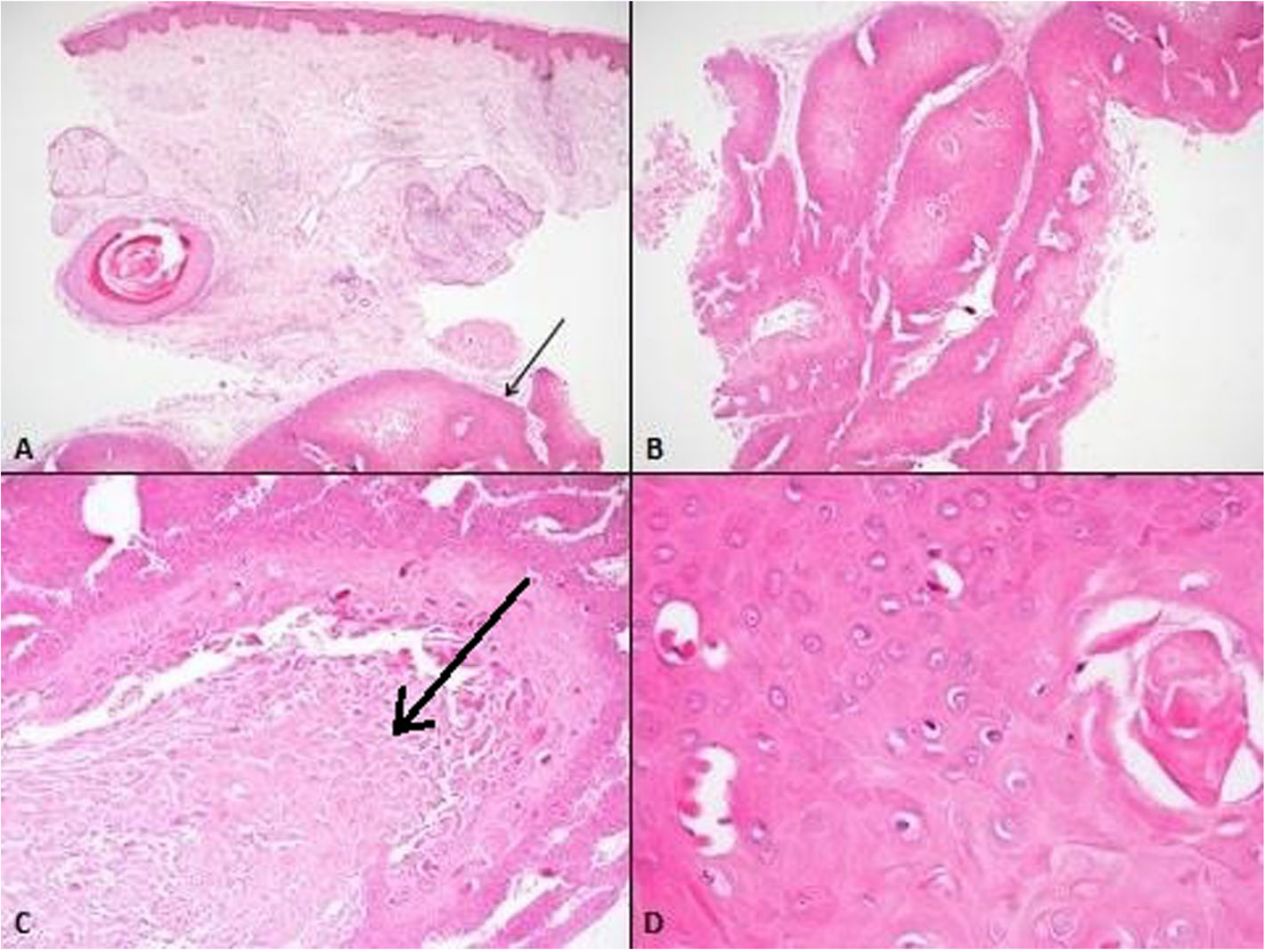
**a** Sections showing a well-circumscribed deep dermal tumor (arrow) (hematoxylin and eosin, × 40). **b** The tumor is composed of expansile lobules of squamous cells separated by loose stroma (hematoxylin and eosin, × 400). **c** There was evidence of abrupt keratinization in the center of some of the lobules, and ghost cells (arrows) are also seen (hematoxylin and eosin, × 100). **d** The cells did not show any atypia or mitotic activity (hematoxylin and eosin, × 400)

**Fig. 2:**
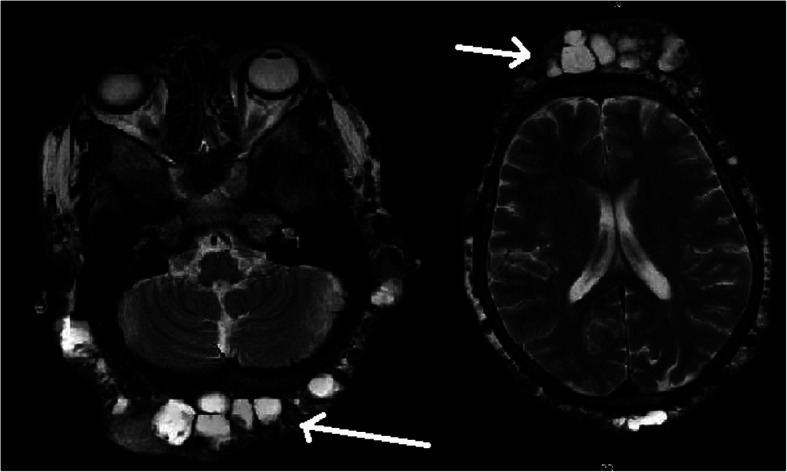
T2-weighted axial MRI images of the brain showing multiple confluent and discrete solid cystic lesions of trichilemmal tumor of the scalp (arrows)

**Fig. 3:**
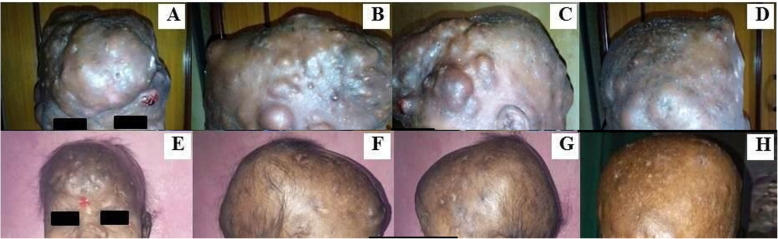
Clinical photograph showing pre and post treatment (6 months) images

A 26-year-old female presented in 2008 with a hyper-pigmented nodule over the scalp which was excised by surgeons. The post-operative histopathology report showed features of a trichilemmal cyst. Over the course of the next 7 years, she developed 3 recurrences in the same site and underwent excision with histopathology revealing trichilemmal cysts. However, in 2017, the patient developed a rapidly enlarging scalp recurrence. Most concerningly, this lesion, unlike prior recurrences, would bleed with only minor trauma. The patient was then referred to the apex government oncologic center. She did not have any other medical co-morbidities. She denied both a personal and a family history of malignancy. She had not been exposed to therapeutic levels of radiation previously. On examination, the patient had an Eastern Cooperative Oncology Group (ECOG) performance status of 1. She was adequately nourished with no pallor or icterus. She had multiple exophytic nodular swellings with overlying pustules which were firm in consistency over the frontal region, vertex, and occipital region of the scalp. Clinical skin examination did not reveal lesions elsewhere. No palpable pre-auricular, post-auricular, occipital, or neck nodes were present. No hepatomegaly or splenomegaly was found. Skin biopsy from the scalp lesion was performed. Sections of biopsied lesions revealed a well-circumscribed deep dermal tumor composed of expansile lobules of squamous cells separated by loose stroma on hematoxylin and eosin (H&E) stain. There was evidence of abrupt keratinization in the center of some of the lobules. The cells did not show any atypia or mitotic activity. Few ghost cells were also seen. A pathological diagnosis of proliferating trichilemmal tumor was made (Fig. 1). Magnetic resonance imaging (MRI) revealed multiple homogenously enhancing confluent and discrete solid cystic lesions in T2-weighted images with signal voids showing blooming on gradient recalled echo (GRE) images within superficial soft tissues of left pre-auricular, temporal, right superficial parotid, sub-occipital, cervical, and scalp regions. The T2-weighted iso-intense solid component was seen interposed between cystic components rather than within cystic components, and both were seen merging with each other. Peripheral enhancement of cystic lesions and solid heterogeneous enhancement of intervening soft tissue component were seen. No intracranial extension was seen. No calvarial destruction or marrow edema was seen (Fig. 2). The patient was deemed inoperable due to the extensive nature of the disease. Instead, she was treated with scalp radiotherapy of 50 Gy in 25 fractions over 5 weeks by volumetric modulated arc technique (VMAT) with a single complete arc of 6 MV photons. The gross tumor volume (GTV) was delineated with the help of pre-treatment MRI and simulation CT. An isotropic margin of 5 mm was outlined around GTV to form the planning target volume (PTV). A 1-cm wax bolus was used over the scalp to pull the isodose curves superficially and provide adequate dose to the scalp. Doses to organs at risk (Table 1) are depicted. The patient tolerated the treatment well with no grade 2 or higher toxicity. At 1 month after treatment, bleeding had ceased and there was greater than 90% reduction in pain. At 6 months after treatment, the nodules had remarkably reduced in size providing a good cosmesis and the patient no longer endorsed pain over the scalp (Fig. 3). The patient has been kept on quarterly (every 3 months) follow-up, and she has been advised to have yearly functional testing of her pituitary gland.

**Table 1:** Dose coverage to PTV and OAR doses

Target	Coverage	Achieved
PTV	95% PTV to receive 47.5 Gy	92% PTV received 47.5 Gy
GTV	100% PTV to receive 47.5 Gy	96% GTV received 47.5 Gy
OARs	Constraints	Achieved
Right eye	Dmean < 35 Gy	12.1 Gy
Left eye	Dmean < 35 Gy	11 Gy
Right cochlea	Dmax < 45 Gy	24.3 Gy
Left cochlea	Dmax < 45 Gy	10.27 Gy
Right optic nerve	Dmax < 54 Gy	9.7 Gy
Left optic nerve	Dmax < 54 Gy	9.97 Gy
Optic chiasma	Dmax < 54 Gy	9 Gy
Right temporal lobe	Dmax < 55 Gy	51.8 Gy
Left temporal lobe	Dmax < 55 Gy	49.7 Gy
Brain	As low as reasonably achievable	Dmax 50 Gy
Brainstem	Dmax < 54 Gy	12 Gy
Spinal cord	Dmax < 45 Gy	26.4 Gy
Right parotid	Dmean < 26 Gy	27 Gy
Left parotid	Dmean < 26 Gy	34 Gy

## Discussion

Proliferating trichilemmal tumors are a rare entity that usually arises in the scalp [3]. It occurs commonly after the sixth decade of life [4, 5]. Due to its rarity, it is frequently misdiagnosed as squamous cell carcinoma or sebaceous cyst. The diagnosis is based on histopathology with PTT exhibiting broad bands of proliferating epithelial cells that either surround cystic areas or are interconnected and separated by a generally fibrous, but occasionally sclerosed or cellular stroma [4]. Within the bands, smaller basaloid cells are seen palisading at the periphery; these cells enlarge and become more squamoid as they progress toward the center [4, 6–8]. Surgical excision with margins of at least 1 cm has been the treatment of choice for PTT. The rate of local recurrence in PTT after wide local excision was found to be 3.7% in a meta-analysis [9]. Radiotherapy as a treatment modality has been used in the adjuvant or palliative setting. In our review of the literature, we found only one case report of a PTT treated with radical radiotherapy [1]. In that case report, a 93-year-old male patient with a 5 cm (in the largest dimension) PTT was treated with 45 Gy of radiotherapy over 3 weeks with electrons. The patient had achieved a complete response at 6 months post radiotherapy and continued to have no recurrence at 2 years after treatment. In light of the larger volume of disease, as depicted in Fig. 2, we decided to treat with 50 Gy. In order to adequately cover the tumor, the photon-based VMAT plan (Table 1) required the irradiation of a portion of the brain. As such, we elected to treat at 2 Gy per fraction over 5 weeks, as opposed to the literature case, where they elected to use a hypofractionated approach of 3 Gy over 3 weeks. The patient tolerated our treatment well with only grade 1 dermatitis and conjunctivitis. The reactions settled within 2 weeks of completion of treatment. At 1 month post therapy, the patient had a satisfactory improvement in both pain and bleeding. The objective response was partial as per the response evaluation criteria in solid tumors (RECIST) v1.1 criteria. By 3 months, tumor regression with good cosmesis was achieved. Tumor regression continued 6 months after treatment. At the time of writing this case report, the residual disease continues to be stable with no progression in size or number of lesions. A theoretical risk of radiotherapy, and one that must be explained to all patients being offered treatment, is malignant degeneration of the tumor. PTTs exist on a spectrum, ranging from relatively benign, such as PTTs that may locally recur but that do not metastasis, to the more aggressive MPTTs that can have significant metastatic potential. In addition, malignant transformation of a PTT into a trichilemmal carcinoma or the development of a radiation-induced malignancy, such as squamous cell carcinoma, is possible and also needs to be discussed. The extent of the tumor, the age of the patient, the importance of cosmesis at the site of disease, and alternative treatments, such as surgery, will all factor into this discussion and the patient’s ultimate decision regarding therapy.

## Conclusion

Proliferating trichilemmal tumors are a rare entity usually treated effectively with surgery. They exist on a spectrum from the relatively benign PTTs that can locally recur but do not metastasize, to malignant PTTs that both can recur and metastasize. The patient was a young unmarried female with extensive disease of her scalp. This report corroborates a prior case report showing good local disease control, tumor regression, and symptomatic improvement which can be achieved with radical radiotherapy. Importantly, when combined with this case report, there is now evidence that both men and women, both young and old, and both focal and diffuse disease may benefit from radiotherapy. In our study, radiation was given at a dose of 50 Gy over 5 weeks. This study reinforces the potential role of radiotherapy in the management of PTTs, especially when the disease is unresectable and involves cosmetically important structures. Radiotherapy is a promising tool in the management of PTTs but, like all treatment modalities, requires informed consent with a comprehensive discussion of its unique potential benefits and risks to the patient.

## Data Availability

Not applicable.

## Abbreviations

PTT: Proliferating trichilemmal tumor
ECOG: Eastern cooperative oncology group
H&E: Hematoxylin and eosin
MRI: Magnetic resonance imaging
GRE: Gradient recalled echo
GTV: Gross tumor volume
PTV: Planning target volume
VMAT: Volumetric modulated arc therapy
RECIST: Response evaluation criteria in solid tumors
